# Annotations

**Published:** 1894-07-07

**Authors:** 


### Annotations.

### A Check upon Public Poisoning.

The case of Oakley v. Leney, which was tried before Mr. Justice Cave few days ago, is one of great interest to dwellers in town and country alike. The point at issue was whether a proprietor of offensive manure has the right to deposit his offensive manure in such a situation as will render it dangerous to the health of other people. Mr. Leney, the defendant in the case, is the tenant of a farm at Chilham, near Canterbury, and whilst cultivating the land himself he sub-let the farm-house to a Mr. Oakley. Soon after Mr. Oakley had entered upon the tenancy of the house, Mr. Leney deposited a large heap of London manure within a distance of 88 yards from the building. Whereupon Mrs. Oakley, the servants, and other persons became ill with sore throats and similar troubles; visitors to the house complained of unendurable odours; and one witness described the smells as "horrible, offensive, dreadful, and abominable." Of course, the usual rebutting evidence was called, and we regret to say that medical experts also, as usual, figured on opposite sides. The Judge, however, summed up with a judicious mixture of science and common sense. According to the germ theory, he said, blood-poisoning was mostly caused by germs found in air, water, milk, and so forth. He believed that the air ot the neighbourhood had been poisoned by the manure, and that the illness of Mrs. Oakley and the other persons concerned had been caused by such poisoning of the air. That kind of thing could not be permitted; and the defendant must pay £150 damages, with costs. In our opinion this decision was thoroughly sound. No doubt farmers must have manure; but it is the easiest thing in the world to deposit any quantity of manure in shallow pits, and to cover it over with earth; or, if it must be placed on the surface of the ground, the mere covering with a thin layer of earth, as large masses of potatoes and other roots are covered for preservation every year, is amply sufficient to prevent smells. In any case, this decision has established the rule that neither farmers nor anybody else may poison the public with vile odours for their own convenience.

### "That Vile Indigestion."

"That vile indigestion, like the poor, seems as if it would be always with us," said a patient to his physician the other day. "What are you doctors doing that you cannot cure it?" The patient was suffering from one of the commonest of all forms of indigestion, and one of the most curable—nervous dyspepsia from overwork and worry. Why, then, was he not cured? The truth is, that he had been cured from time to time, but his trouble had recurred with the recurrence of his over-work and worry. But why could he not be cured permanently? For the very simple reason that doctors, like other people, cannot make bricks without straw, cannot drive engines without steam, cannot fly without wings, or do any other thing which science and common sense both declare to be impossible. We have hundreds of methods of successful cure, but some of our patients are too poor to employ them, some are too busy getting rich, some are too weak-minded to perseveringly carry out our orders. If doctors could always cure by the mere prescribing of a bottle, or a powder, or a pill, they would be conjurers, not men of science. The doctor can only cure by restoring to its natural condition that organ or function which circumstances have disordered. If the patient cannot, or will not, take the needed relief from overstrain, or otherwise modify his circumstances in addition to the use of medicine, how can the cure be effected? You cannot both build up and pull down a house at the same moment. Doctors would work many wonders which are now left unworked if all their patients were rich and all were reasonable.

### Commercial Medicine: A Prophecy Fulfilled.

Three weeks ago, when discussing the question of medical aid societies, we ventured to assert the probability that in no long time "specialisms" in medicine would be taken up by syndicates of laymen or others, and run as commercial speculations. The fulfilment of our quasi-prediction has come sooner than we anticipated. The *Financial News* for Wednesday, June 27th, 1894, published several columns under the heading of "New Companies Registered," and among the rest was announced "Ware's Eczema and Anæmia Cure Company (Limited)." The note in the *Financial News* states that the company is "Registered by W. G. Reader and Co., 7, Ely Place, W.C., with a capital of £8,000 in £1 shares. Object, to adopt and carry into effect an agreement, made June 16th, 1894, between J. Ware on the one part, and C. Major of the other part, and to manufacture and deal in remedies for eczema and other skin diseases, and anæmia. Table A mainly applies." Every intelligent medical man knows that eczema, and practically all skin diseases, are not diseases of the skin merely, but diseases of the general system, which show themselves chiefly in the skin, and that, moreover, they require for their safe treatment just as much scientific knowledge and trained capacity as diseases of the internal organs. Anæmia, a symptom accompanying a great variety of pathological conditions, demands for its safe and successful treatment, not only the common knowledge of the average practitioner, but very frequently special capacity and special experience. Yet these are the diseases, or symptoms of diseases, which are to be treated wholesale by the ordinary methods of commercial enterprise. We see here a deadly blow aimed at honest medical science and progress. We see the beginning of a downward rush, commenced by outsiders, but which can hardly fail to be followed in self-defence by practitioners who have hitherto adopted the methods of plain and honest simplicity in their practice. Most assuredly if the legislature were wise it would firmly put down its foot and stamp out such methods of practice at once. But, if the medical profession is to be what it ought to be, the guardian and conservator of a true medical science and of the health and happiness of the public, it, at any rate, will promptly take effective steps to suppress at the beginning such dangerous departures as we here make public. As we said before, if capital is once imported on a considerable scale, and large vested interests are once created, the medical profession will find itself absolutely powerless to prevent the wholesale degradation of medical science by ignorant traders. It must be "now or never" if anything effective is to be done.

